# Cardio-metabolic and socio-environmental correlates of waist-to-height ratio in German primary schoolchildren: a cross-sectional exploration

**DOI:** 10.1186/s12889-018-5174-6

**Published:** 2018-02-23

**Authors:** Eva Vorwieger, Anne Kelso, Jürgen Michael Steinacker, Dorothea Kesztyüs, on behalf of the URMEL-ICE study group

**Affiliations:** 1 Division of Sports and Rehabilitation Medicine, Ulm University Medical Center, 89075 Ulm, Germany; 2 Institute of General Medicine, Ulm University, Helmholtzstraße 20, 89081 Ulm, Germany

**Keywords:** (MeSH), Abdominal obesity, Etiology, Prevention & control, Child, Blood pressure, Lipids

## Abstract

**Background:**

Controversial messages of childhood obesity emerge: Levelling off in terms of body mass index (BMI) is foiled by increases in abdominal obesity. Waist-to-height ratio (WHtR) may be used as a screening tool for abdominal obesity in children. The aim of this study was to investigate clinical and socio-environmental correlates of abdominal obesity in primary schoolchildren.

**Methods:**

Cross-sectional data from 753 children participating in baseline assessments of the outcome evaluation of a school-based prevention program were analysed. Abdominal obesity was defined as WHtR ≥0.5. According to German age and sex-specific BMI-percentiles, overweight (>90th percentile) and obesity (>97th percentile) were determined. Anthropometric and sonographic measurements, blood pressure and blood samples were taken by clinical staff in a standardized manner. Socio-environmental and lifestyle data were assessed via parental questionnaires. Differences between abdominally obese children and others, and correlations of WHtR with clinical data were tested. Socio-environmental correlates of abdominal obesity were explored in a logistic regression analysis.

**Results:**

At the time of the examination children were 7.57 ± 0.42 years old. Abdominal obesity was observed in 132 (17.5%) children. According to BMI-percentiles, 22.9% of these children were obese, 38.2% overweight, and 38.2% normal weight. Affected children more often used screen media and less often participated in club sports. Abdominal obesity was associated with higher blood pressure, lower HDL- and higher LDL-cholesterol. WHtR significantly correlated with intra-abdominal fat thickness (IAF). The logistic regression model revealed migration background (odds ratio (OR) 2.12, 95% confidence interval (CI) [1.41, 3.19]), smoking during pregnancy (OR 2.30, 95% CI [1.37, 3.86]), parental obesity (OR 1.95, 95% CI [1.22, 3.10]) and higher educational level (OR 0.64, 95% CI [0.42, 0.98]) to be significantly associated with abdominal obesity in children.

**Conclusion:**

WHtR correlates strongly with IAF. Abdominal obesity in primary schoolchildren is associated with cardio-metabolic risk factors and also occurs in otherwise normal weight children. Against the background of rising numbers of abdominal obesity in children, targeted preventive measures are long overdue. The focus of such measures should be used on children with migration background and involve parents, especially those who are obese and those with lower educational levels.

## Background

Despite an observed levelling off in the numbers of children affected by obesity, based on the body mass index (BMI) [1, 2], other researchers report continuously rising numbers of abdominal obesity [3, 4]. Moreover, BMI obviously fails to correctly classify about a quarter of obese children [5]. A change in awareness towards the importance of abdominal obesity is long overdue. The health risks of obesity are evidenced by abdominal fat accumulation [6], and abdominal obesity is very likely to be carried forward from childhood to adolescence [7]. Abdominally obese children already have higher rates of absence from school and more visits to a physician [8]. In children as well as in adults, the presence of abdominal obesity is an important part of the definition of the metabolic syndrome, accompanied by disturbances of blood pressure, blood lipids and blood glucose or insulin [9]. Individuals diagnosed with the metabolic syndrome are at high risk for type 2 diabetes and cardiovascular disease (CVD) [10]. But abdominal obesity is not only associated with type 2 diabetes and CVD, newer research shows emerging evidence of a link between visceral adiposity and carcinogenesis [11]. Furthermore, abdominal obesity negatively affects the respiratory system mechanics and may worsen lung function [12]. Abdominal obesity may occur in otherwise normal weight individuals and is associated with a higher mortality risk [13]. In the light of the worldwide rise of non-communicable diseases (NCDs) [14], and the threat they pose to public health and national economies [15], more research needs to be done to determine, how early health-related correlates of abdominal obesity emerge. Additionally, socio-environmental correlates are of interest to focus targeted prevention on vulnerable groups.

Recent research has identified several correlates of abdominal obesity in children. Skipping breakfast seems to be a risk factor in general for the development of overweight and obesity in childhood [16], and may also lead to abdominal obesity [17, 18]. Other factors that have been identified are chronic sleep curtailment [19], stress, lack of physical activity, and positive energy balance [20]. Maternal smoking during pregnancy and parents who currently smoke are discussed as further risk factors [17, 21]. Parental weight is often correlated with abdominal obesity in children [17, 22]. Socio-economic factors like low household income may also correlate with abdominal fat accumulation in youths [17].

Visceral fat accumulation is associated with an altered metabolic profile in adults [23]. In a Norwegian study on physical activity among children, waist circumference (WC) was found to be related to low-grade inflammation in 9- and 15-year-olds [24]. Abdominally obese children were found to have more likely adverse levels of LDL and HDL cholesterol, triglycerides, and insulin in the Bogalusa Heart Study [25]. Furthermore, blood pressure was positively associated with large WC in a cross-sectional sample of 12-year-olds in the Netherlands [26]. Waist-to-height ratio (WHtR) as a measure of abdominal obesity offers several advantages over WC and BMI, and is increasingly recognized as a screening tool for individual health risks [27]. WHtR is also applicable in children and does not depend on age and sex-specific percentiles [28].

The aim of the present study was to identify primary schoolchildren with abdominal obesity (WHtR ≥0.5) and to explore associated clinical factors like blood pressure, blood lipids and intra-abdominal fat thickness, and further determinants of lifestyle and socio-environmental environment.

## Methods

### Study design

As a part of the school-based lifestyle intervention programme “URMEL-ICE (Ulm Research on Metabolism, Exercise and Lifestyle in Children)” a baseline, cross-sectional analysis was carried out to examine cardio-metabolic and socio-environmental correlates of abdominal obesity in primary school children in southwest Germany. The study was approved in 2006 by the ethics committee of Ulm University and conducted according to the Declaration of Helsinki. Written informed consent was obtained from the parents. Prior to each examination, the oral consent of the child was obtained. More detailed information has already been published elsewhere [29].

### Participants and data

Data was collected from May to October 2006. Baseline data from 753 children (age range 6.3–9.2 years) was included in this analysis, representing a sub-sample of the overall sample of 1119 children who participated in the URMEL-ICE intervention. Only those children with complete information on WHtR and clinical parameters, except ultrasonic measurements, were included in this analysis.

### Anthropometric measurements

Anthropometric measurements were performed at the paediatric clinic at the Ulm University Medical Centre by trained staff. Body height was measured to the nearest 0.1 cm (Ulmer Stadiometer, Busse Design Ulm GmbH, Ulm, Germany). Children were barefoot and advised to stand in an upright position. Body weight was measured to the nearest 0.1 kg using an electronic scale with the child standing barefoot on the scale and wearing only underwear (SECA 701, Seca Hamburg, Germany). The BMI was calculated (kg/m^2^) and converted to sex and age-specific BMI-percentiles (BMIPCT) according to German reference values [30]. Overweight and obesity was defined for values above the 90th and the 97th percentile, respectively, the cut-off for underweight was the 10th percentile.

Waist circumference was measured twice to the nearest 0.1 cm at level of the umbilicus using an executive diameter tape (Seca, Hamburg, Germany). The average of the two measurements was calculated and used for further analysis. Abdominal obesity was defined as WHtR ≥0.5 [28].

Parental BMI was calculated according to self-reported height and weight and categorised as overweight (BMI > 25.0) and obese (BMI > 30.0), according to the international classification of the World Health Organization (WHO) [31].

### Clinical measurements

Blood pressure was measured on the right arm using an electronic monitor (1846 SX Vital Data Monitor, Critikon-Dinamap, Tampa, Florida, USA) with the child laying in a supine position and the upper body elevated up to 30%. The cuff was selected according to the arm circumference (small adult 17–25 cm or adult 25-35 cm) and fixed at the level of the heart. Blood pressure was measured twice and a mean value calculated.

Venous blood samples were taken at the crook of the arm. About 30 min prior to blood collection, an anaesthetic cream was applied to the injection site (EMLA Crème, AstraZeneca GmbH, Wedel, Germany). Blood samples were collected throughout the morning, which means not all samples were taken from children with an empty stomach. However, this is not important for determining cholesterol values. The cholesterol analysis was performed at the laboratory of chemical science at the Ulm University Medical Centre. High density (HDL) and low density (LDL) lipoprotein and total cholesterol were measured using the CHOLESTECH-LDX analyser (MICRO-MEDICAL Instrumente GmbH, Königstein/Taunus, Germany).

The ultrasonic measurement of intra-abdominal fat thickness (IAF) was performed using Philips HD11XE (Philips Medizin Systeme GmbH, Hamburg, Germany) with a C5–2 transducer (Philips Medizin GmbH, Hamburg Germany). IAF is defined as the distance between the posterior wall of the musculus rectus abdominis and the anterior wall of the aorta at the branching of the arteria mesenterica superior [29].

Intima media thickness was also determined via ultrasound (Philips HD11XE, Philips Medizin Systeme GmbH, Hamburg, Germany) using a 12–3 MHz linear transducer (Philips Medizin GmbH, Hamburg Germany). The measurement was performed with the child laying in a supine position, the upper body exposed and the neck elongated. The left (common) carotid artery was measured at about 1 cm before its branching into the internal and external carotid artery. A video recording of the pulsating artery was used to automatically measure the end-diastolic thickness of the intima-media-complex. This measurement was performed by two specialists and repeated twice. Measurement accuracy is 0.01 mm.

### Socio-environmental characteristics

Information on socio-environmental characteristics was assessed by means of a self-administered parental questionnaire. Parental variables included maternal smoking during pregnancy, breastfeeding of the offspring, single parenthood and the parents` level of education. Lifestyle characteristics of the children included breakfast habits, consumption of sweetened drinks, screen media time and frequency of participation in club sports and non-club sports. Migration background was queried in the parental questionnaire and is defined as either the child’s mother or father being born outside of Germany and/or if one of the parents spoke a foreign language during the child’s first years of life.

### Statistical analysis

Differences between children who were abdominally obese and those who were not, were tested applying Fisher’s exact test for categorical data and the Mann-Whitney U-test for continuous data. Correlates of WHtR with clinical parameters were tested according to the underlying distributions with Pearson’s product moment correlation coefficient or Spearman’s rank-order correlation coefficient.

All variables characterizing social environment and lifestyle were further included into a logistic regression model in order to confirm the association and to obtain adjusted measurements. To account for the clustering of data in schools, a possible school-effect was examined in a generalized linear mixed model.

Variables with missing values in the final regression model were imputed five times using the multiple imputation procedure within SPSS. The complete case logistic regression model is reported alongside the combined multiple imputation logistic regression model.

All above-mentioned analyses were carried out using the statistical software packages IBM SPSS release 21.0 for Windows (SPSS Inc., Chicago, IL, USA) and R release 3.2.3 for Windows (http://cran.r-project.org). Sample size in the analyses may vary due to some missing data. The significance level was set at α = 0.05 for two-sided tests.

## Results

### Baseline characteristics

Data from 753 children with complete information on blood pressure, blood lipid values, and abdominal obesity were available for analyses. The children were 7.57 ± 0.4 years old, and 54.3% were boys. The overall percentage of abdominal obesity was 17.5%, divided into 38.2% of normal weight and 61.1% of overweight and obese children. Comparing those with abdominal obesity to those without, the former more often had a migration background, were overweight or obese and less often normal weight or underweight. Children with abdominal obesity showed a higher IAF, higher systolic blood pressure, lower HDL and higher LDL as their lean counterparts. Mothers of abdominally obese children had more often smoked during pregnancy. Parents were more likely to be less educated or to be obese. Those children who were abdominally obese spent more time with screen media and participated less often in club sports. See Table 1 and Table 2 for further information.

**Table 1:** Baseline characteristics of participants in the URMEL-ICE study

	Missing Values	WHtR ≥0.5 (*n* = 132)	WHtR < 0.5 (*n* = 621)	Total (*n* = 753)
Boys, n (%)		65 (49.2)	344 (55.4)	409 (54.3)
Age, years [m (sd)]		7.57 (0.42)	7.57 (0.41)	7.57 (0.42)
Migration background, n (%)		66 (50.0)***	179 (28.8)	245 (32.5)
Parental characteristics				
Maternal smoking during pregnancy, n (%)	9	31 (23.7)**	71 (11.6)	102 (13.7)
Breastfeeding, n (%)	18	99 (78.0)	500 (82.2)	599 (81.5)
Single parent, n (%)	5	15 (11.5)	66 (10.7)	81 (10.8)
At least one parent educated > 10 years, n (%)		41 (31.1)***	288 (46.4)	329 (43.7)
At least one parent obese, n (%)	57	38 (30.9)***	92 (16.1)	130 (18.7)
Children’s lifestyle characteristics				
No breakfast before school, n (%)	10	23 (17.8)	75 (12.2)	98 (13.2)
Consumption of sweetened drinks > 2/week, n (%)	59	34 (29.6)	143 (24.7)	177 (25.5)
Screen media ≥1 h/day, n (%)	12	73 (57.0)**	245 (40.0)	318 (42.9)
Playing outside > 2 times a week, n (%)	16	119 (94.4)	588 (96.2)	707 (95.9)
Club sports > 2 times a week, n (%)	35	8 (6.5)*	76 (12.8)	84 (11.7)
Non-club sports > 2 times a week, n (%)	76	29 (25.4)	188 (33.4)	217 (32.1)

*m* mean, *sd* standard deviation

*p < 0.05, **p < 0.01, ***p < 0.001

**Table 2:** Anthropometry and clinical parameters of participating children in the URMEL-ICE study

	Missing Values	WHtR ≥0.5 (*n* = 132)	WHtR < 0.5 (*n* = 621)	Total (*n* = 753)
Anthropometry				
Underweight, n (%)	6	1 (0.8)***	66 (10.7)	67 (9.0)
Normal weight, n (%)	6	50 (38.2)***	543 (88.1)	593 (79.4)
Overweight, n (%)	6	50 (38.2)***	5 (0.8)	55 (7.3)
Obese, n (%)	6	30 (22.9)***	2 (0.3)	32 (4.3)
WC, cm [m, (sd)]		70.14 (5.88)***	57.20 (3.83)	59.49 (6.62)
WHtR, [m, (sd)]		0.54 (0.04)***	0.45 (0.02)	0.47 (0.04)
Clinical parameters				
Intra-abdominal fat thickness, mm [m, (sd)]	10	60.6 (8.0)***	52.1 (6.8)	53.6 (7.7)
Intimia media thickness, mm [m, (sd)]	10	0.44 (0.03)	0.44 (0.03)	0.44 (0.03)
SBP, mmHg [m, (sd)]		113.6 (11.0)***	107.2 (9.8)	108.3 (10.3)
DBP, mmHg [m, (sd)]		61.1 (8.4)	59.6 (7.4)	59.9 (7.7)
SBP or DBP ≥ 90th percentile, n (%)		66 (50.0)***	171 (27.5)	237 (31.5)
Cholesterol, mmol/l [m, (sd)]		4.28 (0.69)	4.21 (0.62)	4.23 (0.36)
HDL cholesterol, mmol/l [m, (sd)]		1.28 (0.24)***	1.42 (0.29)	1.40 (0.29)
LDL cholesterol, mmol/l [m, (sd)]		2.58 (0.62)***	2.42 (0.57)	2.45 (0.58)

*m* mean, *sd* standard deviation, *WC* waist circumference, *WHtR* waist-to-height ratio, *SBP* systolic blood pressure, *DBP* diastolic blood pressure, *HDL* high density lipoprotein, *LDL* low density lipoprotein

*p < 0.05, **p < 0.01, ***p < 0.001

### Correlations of WHtR with clinical parameters

Table 3 shows correlation coefficients of clinical parameters with WHtR as a metric variable and with each other. There is a significant positive correlation of WHtR with IAF. WHtR also correlates positively with blood pressure and LDL cholesterol. A negative correlation is seen for WHtR and HDL cholesterol.

**Table 3:** Correlation coefficients of clinical parameters with WHtR and among one another (*n* = 753) in the URMEL-ICE study

	Missing values	WHtR	HDL cholesterol	LDL cholesterol	Systolic blood pressure	Diastolic blood pressure	Intima media thickness
HDL cholesterol		−.18***					
LDL cholesterol		.12**	−.11**				
Systolic blood pressure		.27***	−.02	.04			
Diastolic blood pressure		.07*	.02	−.01	0.54***		
Intima media thickness^a^	10	.03	−.01	−.02	.09*	.09*	
Intra-abdominal fat thickness	10	.49***	−.20***	.02	.05	.05	.10**

^a^Spearman’s correlation coefficient due to non-normality. WHtR (waist-to-height ratio), HDL (high density lipoprotein), LDL (low density lipoprotein)

*p < 0.05, **p < 0.01, ***p < 0.001

**Fig. 1:**
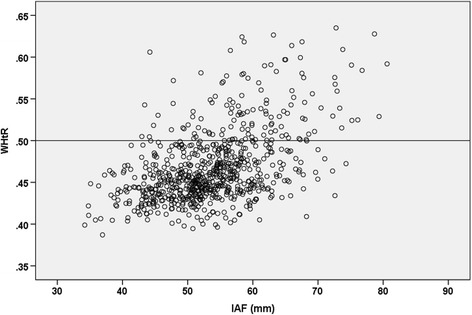
Scatter plot of the correlation between waist-to-height ratio (WHtR) and intra-abdominal fat mass (IAF) in the URMEL-ICE study (*n* = 743). The horizontal line indicates the threshold for abdominal obesity (WHtR ≥0.5)

**Fig. 2:**
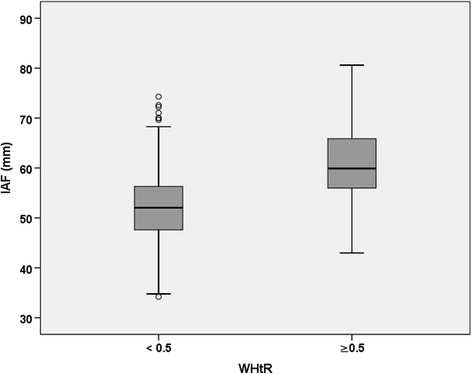
Box-and-whisker plots of the distribution of intra-abdominal fat mass for both categories of waist-to-height ratio (WHtR) in the URMEL-ICE study (*n* = 743)

To visualize the correlation between WHtR and intra-abdominal fat mass, Fig. 1 shows a scatter plot of the two variables. For the association of abdominal obesity (WHtR ≥0.5) with intra-abdominal fat mass, Fig. 2 shows the respective box plots.

### Regression analysis for correlates of abdominal obesity

The final logistic regression model for social, environmental, and lifestyle characteristics associated with abdominal obesity, consists of a migration background of the offspring, maternal smoking during pregnancy, at least one obese parent, and one parent being educated 10 years or longer. Table 4 shows adjusted odds ratios (OR) for the just described variables.

**Table 4:** Unadjusted odds ratios and adjusted odds ratios from logistic regression for WHtR ≥0.5, models with complete cases and multiple imputation in the URMEL-ICE study

		Unadjusted		Complete cases (*n* = 692)		Multiple imputation (*n* = 753)	
	MV	OR	95% CI	OR	95% CI	OR	95% CI
Migration background		2.47***	[1.68, 3.62]	2.12***	[1.41, 3.19]	2.24***	[1.51, 3.31]
Smoking during pregnancy	9	2.37**	[1.48, 3.80]	2.30**	[1.37, 3.86]	2.01**	[1.23, 3.29]
Mother and/or father obese	57	2.34***	[1.50, 3.64]	1.95**	[1.22, 3.10]	1.86**	[1.17, 2.97]
At least one parent educated > 10 years		0.52**	[0.35, 0.78]	0.64*	[0.42, 0.98]	0.64*	[0.42, 0.96]

*MV* missing values, *OR* odds ratio, *CI* confidence interval

*p < 0.05, **p < 0.01, ***p < 0.001

There were no differences seen between ORs and CIs, respectively, in the generalized linear mixed model adjusted for a possible cluster effect in schools, therefore the simple logistic regression model is reported.

## Discussion

In the final regression model, children’s migration background and parental characteristics like obesity, educational level and maternal smoking during pregnancy turned out to correlate with abdominal obesity in primary schoolchildren. Interestingly however, behavioural characteristics of the children, namely screen media use and participation in club sports, primarily significant in the bivariate analysis, could not sustain their significance after being adjusted for those parental characteristics. Furthermore, age and sex had no significant impact on the outcome.

Except for intima media thickness and total cholesterol, all clinical variables correlated with WHtR, though the correlation of diastolic blood pressure was weak. These results underline once more the metabolic meaning of abdominal obesity [23], also in children.

### Correlates and risk factors of childhood abdominal obesity in current research

Children with a migration background are more than twice as likely to be abdominally obese than their peers. A similar proportion of abdominal obesity in migrants was observed in a later study examining primary school children in the whole state of Baden-Württemberg, Germany [8]. This might be due to different nutritional standards and foods as well as other physical activity patterns and a more pronounced sedentary lifestyle. For example, in Germany children and adolescents with a migration background more often consume sweets and soft drinks than the others [32]. In our sample, children with a migration background accumulated obesogenic factors, underlining the vulnerability of this specific group and the importance to focus on them. In comparison to children without a migration background their parents had lower educational levels, were more often obese, mothers had more often smoked during pregnancy, children more often had no breakfast before school, consumed more soft drinks and screen media, and played outside less often.

The child of a mother who smoked during pregnancy has twice the chance to be abdominally obese. This result is in line with many other studies on childhood obesity and maternal smoking during pregnancy [33]. Researchers from Bavaria found a dose-dependent association not explained by confounders and assumed that intrauterine exposure might account for their findings [34].

Obesity in parents is a well-known risk factor for weight problems in their offspring. Due to genetics and parental behaviour, children born to obese parents are at a greater risk to become obese themselves [35]. A cross-sectional study in Brazil confirms our findings for abdominal obesity in children, at least for maternal weight status, as they did not take paternal weight into account [36].

Parental education may have an impact on weight in children because of its association with parental health behaviour and parenting practices. Rodenburg et al. found out that low parental education was associated with an unhealthy cluster of parenting practices such as high visibility and accessibility of screen media and unhealthy food, while higher education was associated with healthier clusters [37].

Missing values are a common problem in studies with observational character and multiple imputation is a recognized method to handle it [38]. We assumed the missing values in the variables migration background and parental obesity were missing at random. Multiple imputations of the missing data changed the values of the logistic regression results only marginally with a slight increase in the impact of migration background and slight decreases for maternal smoking during pregnancy and parental obesity.

In this study, WHtR as a marker for abdominal obesity was positively correlated with IAF, systolic and diastolic blood pressure and LDL-cholesterol. It was negatively correlated with HDL-cholesterol and no correlation was detected for total cholesterol and intima media thickness. The results for blood pressure are similar to other studies of children and adolescents, especially the more pronounced association of systolic blood pressure with abdominal obesity [39]. The association of an abnormal lipid profile with abdominal obesity in youth was documented in some studies [39] and was found to be prevalent even in normal weight abdominally obese individuals [25]. Although abdominal obesity is discussed to be a risk factor for increased carotid intima-media thickness in obese children [40], we could not find this association in our study. A systematic review of adiposity and carotid intima-media thickness in children and adolescents found no association in pre-adolescents, but three studies in adolescents reported correlations of abdominal obesity and carotid intima-media thickness [41]. The strong correlation of WHtR and IAF is also reflected in the regression model, comprising almost the same set of variables as in an earlier analysis, utilizing IAF as the outcome parameter [29].

### Strengths and limitations

The cross-sectional nature of the study does not allow for the assumption of causal associations. The underlying sample is not representative due to selection bias that may have occurred at two levels. Firstly the teachers had to opt in to implement the intervention, and secondly the parents had to give written informed consent for their children to take part in the evaluation. Nonetheless, the response rate was excellent: 78% of eligible participants enrolled in the study. A great deal of the information was assessed via parental questionnaires, which may have led to several restrictions like social desirability bias, reporting and recall bias, measurement bias and missing values.

This study includes a broad variety of recognized and supposed determinants of obesity in primary school children. A special strength of the present research is the accuracy of clinical measurements in view of the large sample size. All children were examined at the Endocrine Outpatient Clinic of Ulm Children’s Hospital. The children’s anthropometric measurements were taken by trained staff according to a pre-specified protocol. The same applies for the measurement of blood pressure and the sonographic assessment of the IAF and the carotic intima-media thickness.

Since the data were collected in 2006, some doubts regarding their topicality may arise. Therefore, we compared the data from 2006 to those of the same age group collected in a large statewide study in Baden-Württemberg in 2011 [42]. This sample does not differ substantially in most, but not all, of the obesogenic variables (migration background, maternal smoking during pregnancy, breastfeeding, single parenthood, parental obesity, no breakfast), but the proportion of children with abdominal obesity was lower in the later sample (10.1% vs. 17.5%). This may be partly due to significant differences in screen media and soft drink consumption between the two samples, both types of behaviour have been addressed during the last years in many German primary schools. Nonetheless, adult Germans are continuously gaining weight, with highest amounts in young men [43], while numbers for children have leveled off since 2006 but are still unacceptably high [44]. Another reason why this research should receive recognition is the large number of relatively young children who took part and who were willing to undergo the at least partly uncomfortable examinations (e.g. blood sampling). More research can be found for older children (aged > 8 years) [45]. Furthermore, in facing the global challenge of overweight and obesity, and the continuing increase in disease burden worldwide [46], any research that confirms existing knowledge and underlines the need for action is valuable. Especially in Germany, adequate political action is not forthcoming despite the growing pressure of obesity and correlated non-communicable diseases.

The generalizability of results is limited, mainly due to selection bias. We suppose that the number of children with abdominal obesity in other parts of Germany may differ. The associations of WHtR with IAF may be transferrable to other children of the same age, as well as associations of WHtR with cardio-metabolic risk factors. The strengths of the correlations of socio-environmental and lifestyle factors with childhood abdominal obesity may vary between groups within the population, but it is generally recognized that obesity is more prevalent in socio-environmentally disadvantaged segments.

### Implications of findings

BMI was developed as a screening tool for different populations [47], and not to determine individual risks. A transition from, focusing mainly on general overweight and obesity as defined by BMI to a more sophisticated look at actual body fat distribution, could lead to a targeted approach towards health risks. Furthermore, especially in children, BMI fails to classify a significant proportion of those who are obese which may be one reason that almost 40% of the abdominally obese children in this research turned out to be of normal weight in terms of BMI. It is high time for paediatricians and health professionals to recognize the limitations of measuring BMI in children and to erroneously rely on respective percentiles when they are about to miss a considerable proportion of children who are at risk for metabolic disturbances. In fact, the highest relative mortality risks in adults were detected for the combination of low BMI with large WC or waist-to-hip ratio (WHR) [48]. Because of the obvious correlation of WHtR with IAF and other markers of cardio-metabolic risk, WHtR may be used as an easily applicable screening tool in children to identify those with a particular need for preventive measures. The analysis of covariates of abdominal obesity shows clearly that preventive measures may not be exclusively implemented for children, but may also need to involve their parents as well. Mothers should be more rigorously informed about the detrimental effects of smoking during pregnancy and more emphasis should be laid on vulnerable groups like migrants and families with lower educational levels. Those vulnerable groups have to be actively involved and supported, for example, by applying a concept of outreach work. Furthermore, preventive measures, as well as therapies for obesity, have to be evaluated for their impact on abdominal girth, with or without weight loss, as some researchers have already proposed [49].

## Conclusion

The present results show significant correlations of abdominal obesity defined as WHtR ≥0.5 in primary schoolchildren with IAF, higher values of blood pressure, higher LDL and lower HDL, which together pose a higher risk for the development of non-communicable diseases, in particular cardio-metabolic diseases. Abdominal obesity occurs also in otherwise normal weight children. The high number of affected children demonstrates the urgent need for appropriate preventive measures. Those measures should predominantly focus on children from vulnerable families with a migration background, or lower educational level. Participation by the parents of these children on a voluntary basis may not be satisfactory, active involvement, e.g. initiated by schools and teachers, is necessary.
